# Characterization of BisI Homologs

**DOI:** 10.3389/fmicb.2021.689929

**Published:** 2021-07-01

**Authors:** Shuang-yong Xu, Elena V. Zemlyanskaya, Danila A. Gonchar, Zhiyi Sun, Peter Weigele, Alexey Fomenkov, Sergey Kh Degtyarev, Richard J. Roberts

**Affiliations:** ^1^New England Biolabs, Inc., Ipswich, MA, United States; ^2^SibEnzyme Ltd., Novosibirsk, Russia

**Keywords:** Type IV restriction system, m5C-dependent restriction endonuclease, BisI family enzymes, thermostable endonuclease, *Bacillus subtilis* T30 methylome

## Abstract

BisI is a sequence-specific and 5-methylcytosine (m5C)-dependent restriction endonuclease (REase), that cleaves the modified DNA sequence Gm5CNGC (G indicates that the cytosine opposite to G is modified). We expressed and purified a number of BisI homologs from sequenced bacterial genomes and used Illumina sequencing to determine the Pam7902I (Esp638I-like) cleavage sites in phage Xp12 DNA. One BisI homolog KpnW2I is EcoBLMcrX-like, cleaving GCNGC/RCNGY/RCNRC sites with m5C. We also cloned and expressed three BisI homologs from metagenome sequences derived from thermophilic sources. One enzyme EsaTMI is active at 37 to 65°C. EsaHLI cleaves GCNGC sites with three to four m5C and is active up to 50°C. In addition, we determined the number and position of m5C in BisI sites for efficient cleavage. BisI cleavage efficiency of GCNGC site is as following: Gm5CNGC (two internal m5C) > Gm5CNGC (one internal m5C) > GCNGm5C (one external m5C) > > GCNGC (unmodified). Three or four m5C in GCNGC site also supports BisI cleavage although partial inhibition was observed on duplex oligos with four m5C. BisI can be used to partially cleave a desired GCNGC site targeted with a complementary oligonucleotide (hemi-methylated). The m5C-dependent BisI variants will be useful for epigenetic research.

## Introduction

In the biological arms race between bacteriophages (phage) and prokaryotic hosts, bacteria and archaea evolved DNA restriction systems (Type I, II, III, and IV) to cleave invading DNA [reviewed in Arber and Linn (1969); Pingoud et al. (2016)]. To counteract the host-encoded Type I-III restriction systems, phages evolved DNA base modifications (e.g., 5-methylcytosines (m5C), 5-hydroxylmethylcytosines (hm5C), 5-β-glucosylated hmC (βghm5C), 5-α-glucosylated hmC (αghm5C), N6-methyladenines (6mA), N6-methylcarbamoyl adenine (mom), N4-methylcytosines (N4mC), or DNA backbone phosphorothioate (PT) modifications) [reviewed in Weigele and Raleigh (2016); Wang et al. (2019)]. In order to attack phage genomes with hypermodified nucleotide (nt) bases, bacterial and archaeal hosts evolved modification-dependent restriction endonucleases (MDREs); MDREs are grouped into Type IIM (e.g., *Dpn*I, G6mATC with defined cleavage) or Type IV restriction systems (e.g., McrBC and GmrSD with undefined cleavage patterns) (Lacks and Greenberg, 1975; Dila et al., 1990; Roberts et al., 2003; He et al., 2015). To simplify the classification it was proposed to include all MDREs into Type IV restriction systems (Weigele and Raleigh, 2016).

BisI restriction endonuclease (REase) was originally found in *Bacillus subtilis* T30; It cleaves modified GCNGC sites in DNA containing two to four m5C/hm5C (http://science.sibenzyme.com/article8_article_7_1.phtml). The genome of *B. subtilis* T30 has been sequenced (Xu et al., 2015). Some BisI homologs were later identified in other bacteria that require two to four m5C/hm5C for efficient cleavage of GCNGC sites (Xu et al., 2016). BisI homologs (e.g., GluI and NhoI) have also been found that require three or four m5C for efficient cleavage of GCNGC sites (Chernukhin et al., 2007; Xu et al., 2016). Other BisI homologs contain additional protein domains. For example, Eco15I and its homologs contain a BisI-like N-terminal domain, and an additional HNH nuclease domain at the C-terminus (Xu et al., 2016). In other hypothetical proteins (ORFs), an N-terminal domain of unknown function is fused to a BisI-like domain at the C-terminus. Other BisI homologs with low amino acid (aa) sequence similarity have evolved into new sequence specificities such as Esp638I (GCNNGC, relaxed site RCNNGY, two to four modified cytosines) (Xu et al., 2016). Thus, studies on BisI homologs may reveal new specificities or new enzymatic properties such as thermostability and faster enzyme turn over. The BisI family of enzymes are unique since they do not share significant sequence similarity with MspJI, McrBC, McrA, EVE-HNH, GmrSD, and PvuRts1I family enzymes, suggesting they evolved independently in evolution to restrict m5C- or hm5C-modified phage DNA.

The goal of this work is to study the rapid evolution of BisI family restriction enzymes in prokaryotes and identified any specificity variants in sequenced microbial genomes and metagenome. Here we examined the *B. subtilis* T30 genome sequence to identify the methylome of this strain, which revealed a Type I methyltransferase (MTase) and a m5C methyltransferase (MTase). We also identified four prophage sequences in the bacterial genome. We characterized BisI homologs enzyme property and DNA recognition sequences as modified variants of GCNGC (RYNRY) and GCNNGC (RYNNRY). In addition, we examined the BisI cleavage efficiency in GCNGC sites containing 1–4 modified cytosines (m5C) and hemi-methylated sites. Finally, we used m5C-modified oligos to hybridize with unmodified double-stranded DNA (dsDNA) and demonstrated partial cleavage of a plasmid DNA. This study broadened our understanding of the BisI family REases.

## Materials and Methods

### Enzymes, Vectors, Phage DNA, and DNA Sequencing

REases, T4 polynucleotide kinase and buffer solutions used in this study were provided by SibEnzyme Ltd. (Russia) or New England Biolabs, Inc. (NEB). Synthetic gene blocks were purchased from Integrated DNA Technologies (IDT). Big-Dye Sanger sequencing kit was purchased from Thermo-Fisher/ABI. Modified DNA substrates for enzyme activity assays are pBR322-*fnu4HIM* (Gm5CNGC, Gm5CNGm5CNGm5C, the cytosine opposite G is also modified) (Xu et al., 2016), T4gt DNA (hm5C), phage Xp12 DNA (m5C). hm5C-containing PCR DNA (2.1 kb) was prepared by PCR amplification from pBR322 using a set of primers, Phusion DNA polymerase and dNTP mix with 5hm-dCTP replacing dCTP (Zymo Research). Phage λ DNA, pBR322, and T4GT7 (unmodified) were used as negative controls.

### Restriction Buffer Compositions (1×)

Buffer 1.1: 10 mM Bis-Tris-Propane-HCl, 10 mM MgCl_2_, 100 μg/ml BSA, pH 7.0 at 25°C. Buffer 2.1: 50 mM NaCl, 10 mM Tris-HCl, 10 mM MgCl_2_, 100 μg/ml BSA, pH 7.9 at 25°C. Buffer 3.1: 100 mM NaCl, 50 mM Tris-HCl, 10 mM MgCl_2_, 100 μg/ml BSA, pH 7.9 at 25°C. CutSmart Buffer: 50 mM Potassium Acetate, 20 mM Tris-acetate, 10 mM Magnesium Acetate, 100 μg/ml BSA, pH 7.9 at 25°C.

### Gene Assembly/Cloning

Synthetic gene blocks were assembled into pTYB1 vector (*Nde*I and *Xho*I digested) using a Gibson assembly kit (NEB) and the DNA inserts were selected by plasmid DNA transformation and the correct insert was verified by DNA sequencing. Plasmids were prepared by plasmid mini-preparation kits from Qiagen or Sigma. Gene blocks encoding Pam7902I catalytic mutants were cloned into pTYB1 expression vector and the inserts were verified by DNA sequencing to contain the desired mutations.

### Protein Expression and Purification

The IMPACT protein expression system including pTYB1 vector and chitin beads was provided by NEB. Target protein was cleaved from the intein-CBD fusion by DTT cleavage overnight and eluted from a chitin column. Enzymes were further purified by chromatography through a heparin column (5 ml HiTrap Heparin HP, GE Healthcare). Proteins were concentrated by low-speed centrifugation in protein concentrators (10 kDa cut-off) and resuspended in enzyme storage buffer (200 mM NaCl, 10 mM Tris-HCl, pH 7.5, 1 mM DTT, 50% glycerol) and kept in a −20°C freezer. Small-scale protein purification was carried out using chitin magnetic beads from 1 ml cell lysate prepared from 20 ml IPTG-induced cells. *E. coli* T7 express (B strain) was used for protein expression. T7 Express cells carrying a restriction gene in a plasmid was cultured at 37°C to late log phase and enzyme production was initiated by addition of 0.5 mM IPTG final concentration and cells were induced at 18 to 20°C overnight in a temperature-controlled shaker. Cells were lysed by sonication in a chitin column buffer (20 mM Tris-HCl, pH 8, 0.5 M NaCl) at 4°C.

### Illumina Library Construction and Sequencing

NEBNext Ultra II DNA library prep kit for Illumina and index primers (set 1) were provided by NEB. To map the cut sites of Pam7902I, phage Xp12 genomic DNA was digested by Pam7902I endonuclease in NEB buffer 2.1. The restricted ends were ligated to the adaptors provided in the library construction kit. Following PCR amplification (15 cycles), the inserts were sequenced using an Illumina sequencing kit (Illumina). Short DNA sequence reads were mapped back to phage Xp12 genome and the cut sites, GCN↓NGC or its variants were deduced using WebLogo (Crooks et al., 2004).

### SMRT Bell Library Construction and SMRT Sequencing

Bacterial genomic DNA (gDNA) was purified from *B. subtilis* T30 cells using the gDNA purification kit purchased from Qiagen. The genome was sequenced using the Pacific Biosciences (PacBio) RSII sequencing platform as previously described (Flusberg et al., 2010; Clark et al., 2012). Briefly, SMRT bell libraries were constructed from a gDNA sample sheared to ∼10–20 kb using the G-tubes protocol (Covaris). The sheared ends were repaired and ligated to PacBio hairpin adapters according to the manufacturer’s protocol. Incompletely formed SMRT bell templates and linear DNA were digested by Exonucleases III and VII (NEB). DNA quantification and the quality of the library was analyzed using the Qubit fluorimeter (Invitrogen) and 2100 Bioanalyzer (Agilent Technology). Two 18-kb SMRT bell libraries were prepared according to PacBio sample preparation protocols for 20 kb libraries and sequenced using C2-P4 chemistry (4 SMRT cells, 120 min collection times). To enhance interpulse duration (IPD) ratios signal, m5C modified bases in the genomic DNA were converted to hm5C by treatment with Tet2 enzyme (catalytic domain, NEB). Tet2 oxidation was carried out according to the protocol for EM-seq kit (NEB, E7120S) (Shen et al., 2018). SMRT library DNA (1 μg) was incubated with Tet2 enzyme in a reaction buffer (50 mM HEPES, pH 8.0, 100 μM Fe(NH_4_)_2_(SO4)_2_, 2 mM ascorbate, 1 mM α-ketoglutarate, 1 mM ATP, 1 mM TCEP) at 37°C for 1 h. Reaction was terminated by addition of stop solution provided in the kit. Software provided by Pacific Biosciences or developed in house was used to detect the modified bases (IPD ratios). An IPD ratio > 1 means that the sequencing DNA polymerase slowed down (relative to the control) at this modified nucleotide position.

SMRT sequencing of *E. coli* genomic DNA modified with M.BisIII expression: *BisIIIM* gene (PCR fragment) was inserted into a T7 expression vector and transferred into *E. coli* T7 Express (a Dam-deficient strain) by transformation. *E. coli* genomic DNA was extracted from overnight cell culture (IPTG-induced) and the gDNA was treated with Tet2 catalytic domain enzyme. The Tet2-treated DNA was used for SMRT sequencing and for detection of CCWGG modified sites based in the IPD ratio.

Modified deoxy-oligoribonucleotide (oligos) substrates were synthesized by SibEnzyme Ltd. (Russia). The sequence and modified base composition of the oligos are shown in Table 1. Duplex DNA oligos were prepared by annealing two complementary oligos which differ from each other by the presence or absence of m5C within the recognition sequence GCNGC and used in BisI digestions.

**TABLE 1:** Modified DNA oligo sequences used in this study.

Name of oligonucleotide	Sequence of oligonucleotide (oligo)
1 (internal m5C)	5′-GCTTGTACTTTA**Gm5CGGC**ATTGATTCTCACCACG-3′
1c	5′-CGTGGTGAGAATCAAT**Gm5CCGC**TAAAGTACAAGC-3′
2 (external m5C)	5′-GCTTGTACTTTA**GCGGm5C**ATTGATTCTCACCACG-3′
2c	5′-CGTGGTGAGAATCAAT**GCCGm5C**TAAAGTACAAGC-3′
3 (two m5C)	5′-GCTTGTACTTTA**Gm5CGGm5C**ATTGATTCTCA CCACG-3′
3c	5′-CGTGGTGAGAATCAAT**Gm5CCGm5C**TAAAGTA CAAGC-3′
4 (unmodified)	5′-GCTTGTACTTTA**GCGGC**ATTGATTCTCACCACG-3′
4c	5′-CGTGGTGAGAATCAAT**GCCGC**TAAAGTACAAGC-3′
N4 (internal N4mC)	5′-GCTTGTACTTTA**G4mCGGC**ATTGATTCTCA CCACG-3′
N4c	5′-CGTGGTGAGAATCAAT**G4mCCGC**TAAAGTACAAGC-3′
P1 (internal m5C)	5′-CATTCAG**Gm5CTGC**GCAACTGTTGGGAAGGGC GATCG-3′
P2	5′-CAGTTGC**Gm5CAGC**CTGAATGGCGAATGGCGCC GATG-3′

*Duplex oligos were prepared by annealing two complementary oligos which differ from each other by the presence or absence of methylcytosine (m5C or N4mC) within the recognition sequence GCNGC.*

### Preparation of the Oligo Substrates for BisI Digestion

The upper strand of each DNA duplex was labeled at the 5′-end using T4 polynucleotide kinase and [γ^32^P]-ATP. After purification with Microspin G-25 columns (Amersham Biosciences UK Limited, England) the ^32^P-labeled oligo was mixed with the complementary unlabeled oligo in a 1:2 ratio. The duplex was then annealed by heating to 95°C for 5 min, followed by slow cooling to room temperature. BisI digestion was carried out in 10 μl of reaction volume containing 1× BisI buffer (10 mM Tris-HCl, pH 9.0 at 25°C, 15 mM MgCl_2_, 150 mM KCl, 1 mM DTT) and duplex oligos at a concentration of 62.5 nM. Reactions were carried out at 37°C for either 1 hour or as indicated.

### Determination of the Cleavage Product Quantities After BisI Digestion

Electrophoresis of the cleavage products was carried out in denaturing 20% polyacrylamide gels and TBE buffer (0.09 M Tris-borate, pH 8.0, 0.002 M EDTA) containing 7 M Urea. Radiolabeled DNA was detected and quantified using a Personal Molecular Imager (Bio-Rad laboratories, Inc., United States). For each labeled product, we determined DLU (Digital Light Units) proportional to the intensity of the [^32^P] isotope radiation minus the background. Percentage of hydrolysis was determined by dividing the reaction product DLU by the total DLU, calculated as the sum of the product DLU and the remaining unhydrolyzed duplex DLU. Data analysis was performed using the program Quantity One – 4.6.2. (Bio-Rad laboratories, Inc., United States).

### BisI Purification and Enzyme Activity Assay

BisI was purified from the lysates of *B. subtilis* T30 cells (grown and frozen previously) by chromatography through three columns (phosphocellulose P11, heparin-sepharose and re-chromatography on phosphocellulose P11). The enzyme preparation was dialyzed and concentrated in a storage buffer (10 mM Tris-HCl, pH 7.55, 0,1 mM EDTA, 0,1 M NaCl, 7 mM β-mercaptoethanol, 50% glycerol) and stored at −20°C. The yield of purified enzyme was estimated to be 6000 Units (U) (2,000 U/ml) from 15 g of frozen cells (see below for BisI unit definition).

To analyze the BisI requirement for m5C in the recognition sequence, four pairs of complementary oligos [1 + 1c (two internal m5C); 2 + 2c (two external m5C); 3 + 3c (four m5C), 4 + 4c (no m5C)] were used (oligo sequences listed in Table 1). We also tested the oligo duplex N4/N4c (two internal N4-methylcytosines, N4mC) to study the possibility of BisI to cleave a DNA substrate containing N4mC. The duplex oligos P1/P2 were used to demonstrate BisI cleavage of plasmid DNA at a pre-determined position. In this case, the pUC19 plasmid pre-linearized with *Dri*I (GACNNN↓NNGTC) at a concentration of 20 μg/ml in TE buffer was mixed with 100-fold excess of P1/P2 oligos (P1 and P2 in equal molar concentrations). The mixture was heated at 95°C for 5 min and then placed on ice. BisI digestion was carried out in BisI buffer at 37°C for 16 h. Reaction products were analyzed by electrophoresis in a 1% agarose gel.

One unit of BisI is defined as the minimum amount of enzyme required for complete digestion of 1 μg plasmid pFsp4HI (Gm5CNGC), pre-linearized with *Bam*HI, in a 50 μl reaction mixture (10 mM Tris-HCl, pH 9.0, 10 mM MgCl_2_, 150 mM KCl, 1mM DTT) at 37°C for 1 h.

BisI activity (in %) for any substrate was determined as the ratio of the tangent in the linear range of their activity curves for the studied substrate compared to the optimal substrate 1/1c.

### Statistical Treatment of Results

Statistical analysis was performed using the method of standard deviation as the statistical test (software Origin 7.0). Microsoft Office Excel 2003 was used for graphical representation of the results.

## Results

### PacBio DNA Sequencing to Determine the Methylome of *B. subtilis* T30 Genome

**FIGURE 1:**
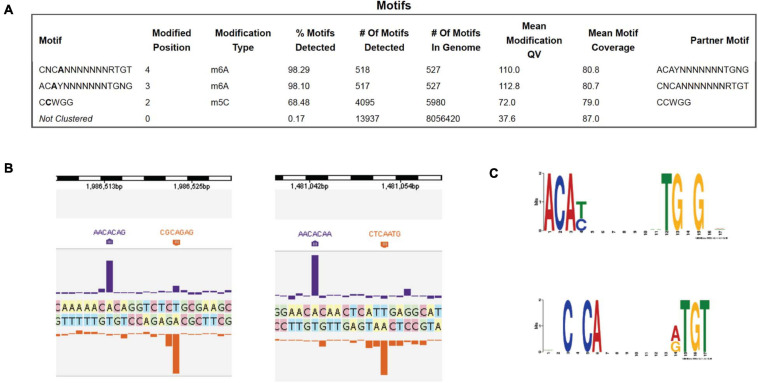
PacBio SMRT sequencing to determine the DNA methylome of *B. subtilis* T30 genome. **(A)** Modified DNA sequence motifs identified by interpulse duration (IPD) ratio and percentage of modified sites: M.BisII (98.1 to 98.3%); M.BisIII (68.5%). **(B)** Two examples of BisII sites modified by M.BisII (6 mA). **(C)** The M.BisII consensus recognition sequence derived from over 500 modified sites. The M.BisII (HsdM/HsdS) target recognition domain TRD1 and TRD2 specificities are ACAY (complement RTGT) and TGNG (complement CNCA), respectively. The 7-bp Type I restriction system is a new specificity.

PacBio SMRT DNA sequencing identified one Type I MTase (M.BisII) with 7 bp specificity CNC6mAN_7_RTGT (complement sequence AC6mAYN_7_TGNG, R = A or G, Y = T or C, N = A, C, G, or T). 98% of the sites in the genome are modified, suggesting constitutive expression of the MTase and S.BisII (Figure 1A). Examples of two modified BisII sites and the consensus sequence derived from over 500 sites are shown in Figures 1B,C, respectively. The M.BisII gene is found in seven other *B. subtilis* strains (genomes) with identical amino acid (aa) sequence. M.BisII also shows 95% aa sequence identity to M.Bcl8716II with the sequence specificity (GNNGAN_7_CTC) which partners with a different S subunit (TRDs) to generate this specificity. More than 950 TRDs have been identified in Type I specificity subunits (HsdS or S) providing a large library from which new restriction specificities could be engineered by TRD shuffling and recombination in partner with the HsdM (M) subunits (REBASE, RJR and Posfai, unpublished result) (Roberts et al., 2015). All known Type I enzymes recognize split sequences of the form CNC6mAN_7_RTGT. There is only one candidate gene for this (*bisIIM* or *hsdM*). Similarly, no Type I enzymes recognize sequences containing m5C, in light of the thousands of examples in the literature as documented in REBASE.

Beside M.BisII, an orphan DNA MTase (M.BisIII, Cm5CWGG) was detected in the genome by SMRT sequencing of Tet2-treated gDNA. A schematic diagram of the location of genes encoding BisI, M.BisII, M.BisIII in the genome is shown in Supplementary Figure 1. Since the specificity of BisI (Gm5CNGC) is quite different from the specificity of M.BisIII (Cm5CWGG), they can co-exist in the same cell and would not be in conflict to cause self-restriction and cell death. Additional evidence was derived from BisI incapability of cleaving Cm5CWGG sites in pBR322 prepared from Dcm^+^ *E. coli* cells (data not shown).

The putative C5 MTase gene next to the BisI restriction gene was also cloned into a T7 expression vector (pAII17). No apparent protein product was detected after IPTG induction in SDS-PAGE analysis (data not shown). Thus, most likely this ORF is an inactive pseudogene. SMRT sequencing also failed to identify additional m5C modified motifs.

### Characterization of Pam7902I Cleavage Site

**FIGURE 2:**
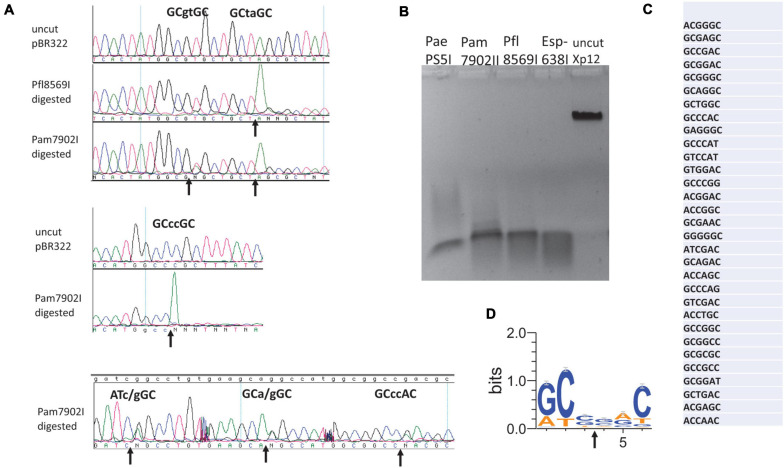
Digestion of modified DNA substrates with Pam7902I and Pfl8569I, DNA run-off sequencing to determine the cleavage sites, and mapping Pam7902I consensus cut sites by Illumina sequencing. **(A)** Examples of cleavage sites by Pam7902I and Pfl8569I digestions and DNA run-off sequencing. The up arrows indicate the nicked template and the appearance of an extra adenine (A, shown in green color) peak after the nick by Taq DNA polymerase (i.e., addition of an adenine by the template-independent terminal DNA transferase activity). **(B)** Restriction digestion of Xp12 DNA (m5C) by PaePS50I, Pam7902I, Pfl8569I, and Esp638I. **(C)** Pam7902I restriction sites (including top 50% of the cleaved sites) digested by Pam7902I and subsequently found in the Illumina DNA library following restriction, library construction and sequencing. **(D)** Consensus DNA sequence (GCS↓SGC or relaxed sites RYS↓SRY) derived from all Pam7902I cleaved sites in Xp12 DNA (compiled by Weblogo).

Esp638I shows a weak aa sequence similarity to BisI (less than 25% sequence identity) and recognizes a different specificity Gm5CS↓SGm5C (relaxed sites Rm5CN↓NGY) (Xu et al., 2016). The recognition sequence can potentially overlap with CpG dinucleotides (e.g., cGCGCGCg). Methylated CpG dinucleotides are important for epigenetic gene regulation, cell differentiation, cancer development and genetic diseases (Hendrich and Bird, 2000). Esp638I homologs are found in many sequenced *Pseudomonas* strains including the human pathogen *P. aeruginosa*. Some *P. fluorescens* strains have biocontrol properties, protecting the roots of some plant species against parasitic fungi. *P. amygdali* is a Gram-negative plant pathogenic bacterium. It is named after its ability to cause disease on almond trees. We cloned and expressed three Esp638I homologs (Pam7902I, Pfl1869I, and PaePS50I. GenBank accession numbers: WP_054068722 (*Pseudomonas amygdali*); WP_049885553.1 (*Pseudomonas aeruginosa*); WP_043042189.1 (*Pseudomonas fluorescens*)) with an emphasis on Pam7902I. The multiple aa sequence alignment is shown in Figure 2A. Esp638I and Pam7902I show 51.8% aa sequence identity and 68.2% aa sequence similarity in pairwise alignment (data not shown). The restriction genes were cloned into pTXB1 (*Nde*I/*Xho*I) and proteins purified from chitin columns by affinity purification, DTT cleavage and elution (data not shown). M.CviPI (GpC methylase)-methylated pBR322 was digested by Pam7902I and Pfl1869I respectively and sequenced by Big-dye Sanger sequencing. Figure 2A shows six cleavage sites conforming to RYNNRY with the best cleavage sites GCCCGC and GCTAGC. In order to look at more cleavage sites, phage Xp12 (m5C) genomic DNA was digested with Pam7902I (Figure 2B) and the restricted fragments were ligated to adaptors in the Illumina library. The sequencing reads were mapped back to the Xp12 genome and the cleavage sites were inferred from the DNA ends. The top 50% of the reads were shown in Figure 2C and the consensus sequence GCSSGC, with occasional degeneracy to RYSSRY, was derived after analysis by WebLogo (Figure 2D). The cleavage site mapping by Illumina library sequencing is consistent with the cut site determined by DNA run-off sequencing of modified plasmid. But the Illumina library sequencing can analyze a few thousand sites and gives a more comprehensive view of the cut-site spectrum.

### Site-Directed Mutagenesis of the Putative Catalytic Site of Pam7902I Endonuclease

**FIGURE 3:**
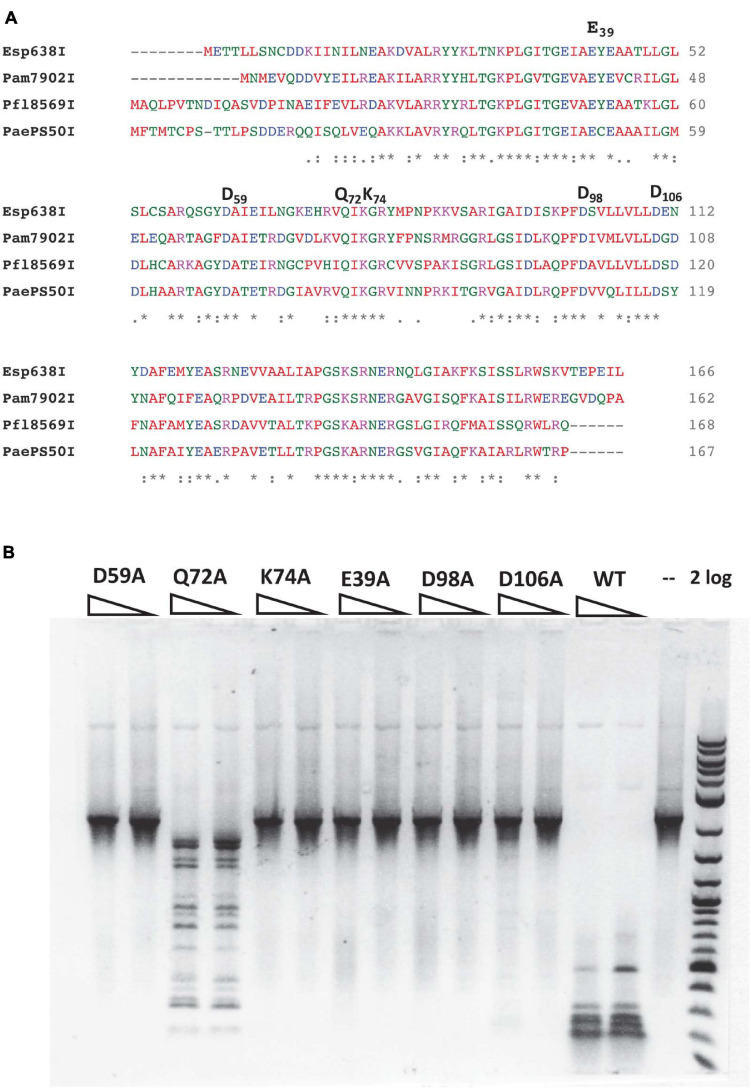
Multiple sequence alignment of Esp638I, Pfl8569I, Pam7902I, and PaePS50I, predicted Mrr-like catalytic site, and activity assay for catalytic mutants. **(A)** Multiple aa sequence alignment by CLUSTAL O (1.2.4). “*”, identical aa residues. “:”, aa residues with similar biochemical property. **(B)** Restriction activity assay for Pam7902I catalytic mutants (two enzyme dilutions). The PCR DNA substrate (2.1 kb) contains modified cytosines-hm5C replacing all cytosines. 2 log, 2-log DNA ladder (0.1 to 10 kb, NEB).

Pam7902I endonuclease contains a Mrr-like catalytic site D-X_(__12__)_-QxK (Orlowski et al., 2008), of which the D59, Q72, and K74 residues may be involved in binding Mg^2+^ cofactor and cleavage of the phosphodiester bond (Figure 3A). To probe the importance of these residues, we changed D59, Q72 and K74 into Alanine (Ala or A) in mutants D59A, Q72A, and K74A. In addition, the conserved negatively charged residues E39, D98, and D106 were also mutated in E39A, D98A, and D106A variants, respectively. The mutant proteins were purified by chitin magnetic beads and DTT cleavage from IPTG-induced cell extracts (data not shown). Figure 3B shows that E39A, D59A, K74A, D98A, and D106A variants abolished the cleavage activity on a hm5C modified PCR DNA. Q72A variant, however, still retains partial cleavage activity. The negative cleavage results of the mutants were also verified on pBR322-Gm5C (M.CviPI-modified) plasmid DNA (data not shown). It was concluded that the predicted catalytic residues D59 and K74 are critical for Pam7902I activity while Q72 is not essential (the divalent cation coordination may be compensated by other negatively charged residues). The conserved aa residues E39, D98, and D106 are also required for Pam7902I activity, but their exact function in modified sequence recognition (binding step) or catalysis remains to be studied.

### Characterization of KpnW2I Restriction Endonuclease

**FIGURE 4:**
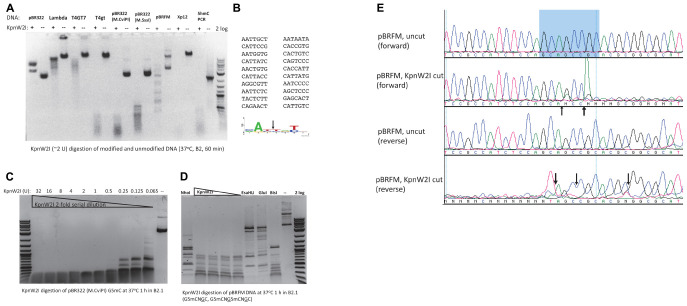
KpnW2I digestion of modified and unmodified DNA substrates. **(A)** Restriction of modified and unmodified DNA. pBR322, phage λ, and T4GT7 were “unmodified”, except Dcm^+^; T4gt and hm5C-PCR DNA contain hm5C; pBR322 (M.CviPI) is m5C-modified at GpC sites; pBR322 (M.SssI) is m5C-modified at CpG sites; pBRFM (pBR322-*fnu4HIM*) is m5C-modified at GCNGC sites; phage Xp12 DNA contains m5C. “ + ” and “–” indicated presence or absence of KpnW2I endonuclease. Two units of KpnW2I were used to digest 0.5 μg of DNA at 37°C for 1 h in B2.1. **(B)** Mapping of KpnW2I nicking sites to RY↓NRY in pBR322. **(C)** KpnW2I enzyme titration on M.CviPI-modified pBR322 (Gm5C). **(D)** Comparison of digestion patterns created by BisI family enzymes NhoI, KpnW2I, EsaHLI, GluI, and BisI. **(E)** Mapping KpnW2I cut site by run-off DNA sequencing. Arrows indicate the cleavage positions.

In a recent BlastP search, we found 80 BisI homologs with 43 to 60% amino acid sequence identity and 17 homologs with 61 to 100% sequence identity (data not shown). KpnW2I shows low sequence similarity to BisI and high sequence similarity to EcoBLMcrX (Rm5CNRC) (Gonchar et al., 2016; Fomenkov et al., 2017). This restriction gene (GenBank accession number WP_064183952) is found in the human pathogen *Klebsiella pneumoniae* W2-1-ERG2. Purified KpnW2I shows a significant amount of DNA nicking activity on pBR322, phage λ, and T4GT7 (unmodified) as shown in Figure 4A. The nicking sites in pBR322 were mapped by sequencing the nicked plasmid. Figure 4B shows the nicking sites at RYNRY. The nicking activity was enhanced in low salt buffer (B1.1) and inhibited in high salt buffer (B3.1) (data not shown). KpnW2I displays higher cleavage activity on modified DNA substrates such as T4gt (hm5C), Xp12 (m5C), M.CviPI-modified (Gm5C) or M.SssI-modified (m5CG) pBR322, pBRFM (Gm5CNGC), or hm5C-containing PCR DNA. The KpnW2I specific activity is estimated to be 8000 U/mg protein as defined as the minimal amount of enzyme for digestion of M.CviPI-modified pBR322 into less than 300 bp fragments for 1 h in B2.1 (Figure 4C). Comparison of KpnW2I cleavage pattern with other BisI family REases show that KpnW2I is the most frequent cutter (Figure 4D). NhoI cuts GCNGC sites with three to four m5C while EsaHLI and GluI prefer to cut GCNGC sites with four m5C (four m5C > three m5C). BisI cuts GCNGC sites with two to four m5C and partially cuts GCNGC sites with one internal m5C. One example of KpnW2I cleavage site is shown in Figure 4E. in which the up and down arrows indicate the cleavage positions (Gm5CAGm5CCGC). The *in vivo* phage restriction activity on modified and unmodified DNA remains to be carried out in a future study. It has been reported previously that some BisI family enzymes (e.g., Bce1273I and Bth17I) display relaxed activity on unmodified DNA (Xu et al., 2016). In some *Klebsiella pneumoniae* strains, a distantly related BisI homolog (GenBank accession number, WP_171996093) is found which has 45% to 47% sequence identity to KpnW2I (data not shown). The m5C-dependent restriction activity of these homologs remains to be investigated.

### Characterization of VarFI

**FIGURE 5:**
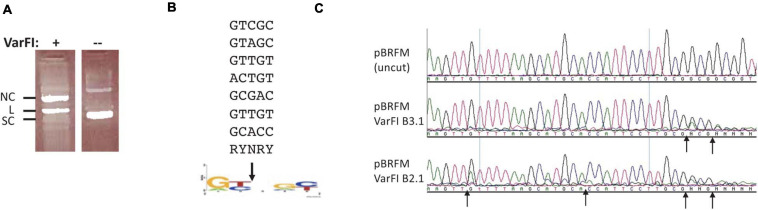
VarFI digestion of pBR322 DNA and mapping of nicking sites in pBR322. **(A)** VarFI digestion of pBR322 (lane 1); lane 2, uncut pBR322. SC, supercoiled DNA; L, linear DNA; NC, nicked circular DNA. **(B)** Consensus sequence (RY↓NRY) of VarFI nicking sites in pBR322. Down arrow indicates the nicked position. **(C)** Mapping of VarFI nicking/cleavage sites by DNA run-off sequencing. The up arrows indicate the nicking/cleavage position of the template strand, where Taq DNA polymerase added an extra A (e.g., A/G or A/C doublet) when the template is broken. Top sequence, pBRFM (uncut); middle sequence, VarFI digested DNA in B3.1; bottom sequence, VarFI digested DNA in B2.1.

VarFI (GenBank accession number ETT82259, *Viridibacillus arenosi* FSL RS-213) shares 53.3% aa sequence identity (71.1% sequence similarity) to BisI and is predicted to be an isoschizomer. VarFI expression in the T7 Express strain was toxic and it proved to be difficult to obtain a clone with correct sequence. Multiple mutants were isolated during the cloning process (data not shown). We cloned the *varFI* restriction gene into pET21b to create a C-terminal 6xHis tag and transformed the vector/insert ligated DNA into a non-T7 strain (NEB Turbo). After sequencing multiple plasmid isolates, one clone with the correct sequence was identified. This clone was then transferred to T7 Express. After IPTG induction, we managed to purify a small amount of protein for enzyme characterization. Figure 5A shows the nicking activity on pBR322 (lane 1). Sequencing the nicked plasmid DNA identified a consensus nicking sites RYNRY (unmodified) (Figure 5B). Figure 5C shows two degenerate nicking sites (GTT↑GT and GCA↑CC) (unmodified) and one modified site (Gm5CG↑Gm5CG↑GC). The nicking activity on unmodified sites was inhibited when the restriction digestion was carried out in a high salt buffer (B3.1) (Figure 3C, middle panel). It was concluded that like KpnW2I, VarFI displays DNA nicking activity on unmodified RYNRY sites and it shows stronger DNA cleavage activity on m5C-modified sites (Gm5CNGC). The toxicity in expression of VarFI may be due to the degenerate nicking activity on unmodified host genomic DNA (VarFI mutants were positively selected during cloning due to loss of activity). It is not known how the original bacterial host deals with this non-specific DNA nicking damage. Presumably, the nicks are quickly re-sealed by bacterial DNA ligase under non-induced condition or the restriction gene expression is tightly repressed. The possible VarFI relaxed specificity (star activity) in the native host, if any, remains to be examined.

### Characterization of RdeR2I Endonuclease

**FIGURE 6:**
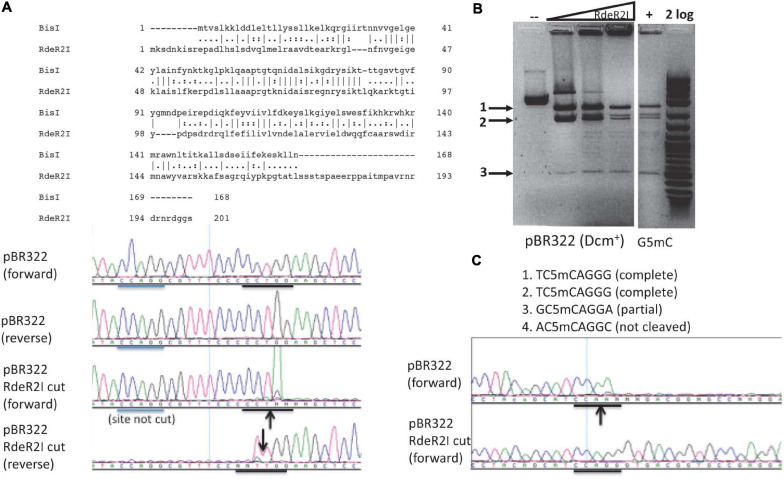
Pairwise sequence alignment between RdeR2I and BisI, RdeR2I activity assay on pBR322 and mapping of cleavage sites. **(A)** Pairwise sequence alignment between RdeR2I and BisI. RdeR2I has an additional 33 residues at the C-terminus. **(B)** RdeR2I activity assay on pBR322 (Dcm^+^) or M.CviPI-modified pBR322 (Gm5C). **(C)** Mapping of the cleavage sites by DNA sequencing indicated that two sites were completely digested (TCCAGGG). The down arrow indicates the strand shown is cleaved (with an extra T peak). The up arrow indicates the opposite strand is cleaved (with the appearance of an extra A peak). Arrows in **(B)** indicate the digested fragments from pBR322, which correspond to the sites shown in **(C)**.

The RdeR2I restriction gene (603 bp, GenBank accession number AGG87590) is found in a *Rhodanobacter denitrificans* strain 2APBS1. It shows 29.8% aa sequence identity and 44.2% sequence similarity to BisI by pairwise sequence alignment (Figure 6A). RdeR2I was partially purified by chitin column chromatography and DTT cleavage. It digested pBR322 (Dcm^+^) DNA (Figure 6B). M.CviPI-modified pBR322 (Gm5C) did not enhance the cleavage efficiency (Figure 6B). Mapping of the cleavage sites by DNA sequencing indicated that two sites were completely digested and one site partially digested (Figure 6C and data not shown). The two completely digested sites have the sequence TCCAGGG and the partially cut site is GCCAGGA. The rest of the Dcm sites were poorly cleaved. We tentatively assigned the RdeR2I specificity as TCm5CAGGG and considered the GCm5CAGGA as a slow star site. This specificity remains to be verified using large m5C/hm5C modified DNA such as Xp12 and T4gt genomic DNA. The recognition sequence of RdeR2I appeared to be quite diverged from BisI. In REBASE, some BisI homologs were predicted to recognize GCWGC sites and have a weak sequence similarity to the TRD of a Type I specificity subunit recognizing GCA.

### Characterization of BisI Homologs From Microbial Metagenomes of Deep-Sea Vent and Hot Springs

**FIGURE 7:**
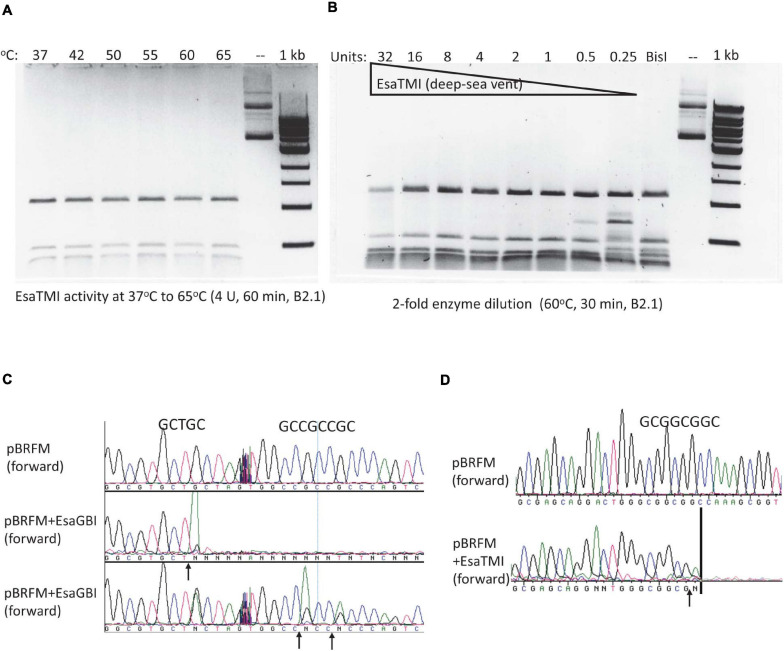
EsaTMI (BisI homolog derived from a deep-sea vent metagenome) restriction activity at various temperatures and EsaTMI enzyme titration. **(A)** Digestion of modified plasmid pBRFM (pBR322-*fnu4HIM*) by EsaTMI (4 U) at 37 to 65°C in B2.1 for 1 h. **(B)** Two-fold serial dilution of EsaTMI in restriction of pBRFM DNA (Gm5CNGC) at 60°C. Over-digestion at 32 U showed some smearing due to star activity. **(C,D)** Cleavage site mapping by DNA sequencing after digestion by EsaGBI and EsaTMI, respectively. The vertical bar in **(D)** indicates that the sequencing peaks are flat after this mark.

By amino acid sequence alignment, we found four BisI homologs from metagenomes derived from deep-sea vent and hot springs microbial sources (Li et al., 2014; Anantharaman et al., 2016). The synthetic gene sequences and amino acid sequences are shown in Supplementary Figure 3. These four genes were synthesized, inserted in pTXB1 and expressed in *E. coli* T7 Express. Three BisI homolog proteins were purified and tested for restriction activity on pBRFM (Gm5CNGC) at a temperature range of 37 to 75°C (one clone failed to produce enough protein for activity assay). EsaTMI (from a deep-sea vent metagenome) is active at 37 to 65°C (Figure 7A) and inactivated at 70–75°C (data not shown). Enzyme titration experiments (2-fold serial dilutions) indicated that EsaTMI displayed star activity at high enzyme concentration (32 U) with DNA smearing (Figure 7B). DNA run-off sequencing results indicated that the enzyme cleaved GCNGC sites containing two or three m5C, a property similar to BisI and Bce95I (Figure 7C). Two other enzymes EsaHLI (from a hot spring metagenome) and EsaGBI (from a deep-sea vent) are active up to 50°C (data not shown). EsaGBI cleaves GCNGC sites with two to three m5C in pBRFM (Figure 7D), while EsaHLI required three m5C in pBRFM for efficient cleavage (data not shown). The number of m5C required for EsaHLI activity is similar to that of SqiI, which cleaves GCNGC sites containing four m5C bases (Xu et al., 2016). EsaHLI shows 98% sequence identity to SqiI and it is anticipated that the m5C requirement is in a similar fashion.

### BisI Digestion of Duplex Oligos With One to Four m5C in GCNGC Sequence

**FIGURE 8:**
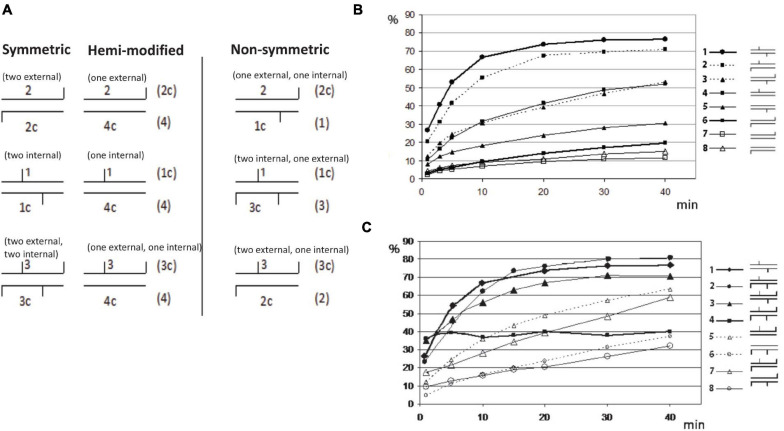
Restriction of GCNGC oligo duplex with one to four m5C. **(A)** Schematic diagram of possible combination of m5C in GCNGC sites. Horizontal lines indicate DNA strands of oligo duplexes, vertical lines indicate m5C as internal (e.g., Gm5CNGC) or external (e.g., GCNGm5C). The names of the corresponding oligos are given above and below each duplex. Alternative variants of oligo duplexes with the same methylation pattern are given on the right in brackets. **(B)** BisI digestion of oligo duplexes with one (hemi-methylated) or two m5C. Percentage of cleavage (%) (*Y* axis) is plotted vs. the time of reaction (*X* axis). Schematic symbols for the methylation type are given on the right: 1 = 1*/1c (two internal m5C, * indicating ^32^P labeled), 2 = 1*/2c (one internal and one external m5C), 3 = 2*/1c (one internal and one external m5C), 4 = 1*/4c (one internal m5C), 5 = 4c*/1 (one internal m5C), 6 = 2c*/2 (two external m5C), 7 = 2c*/4 (one external m5C), 8 = 4*/2c (one external m5C). **(C)** BisI digestion of oligo duplexes with two to four m5C (% of cleavage in *Y* axis is plotted vs the time of reaction (*X* axis)). Schematic symbols of methylation type are given on the right: 1 = 1*/1c (two internal m5C), 2 = 1*/3c (two internal and one external m5C), 3 = 3*/1c (two internal and one external m5C), 4 = 3*/3c (two internal and two external m5C), 5 = 3*/4c (one internal and one external m5C, hemi-methylated on top strand), 6 = 4*/3c (one internal and one external m5C, hemi-methylated on bottom strand), 7 = 3*/2c (one internal and two external m5C), 8 = 2*/3c (one internal and two external m5C).

It was shown previously that some BisI-like enzymes can efficiently digest GCNGC sites with two m5C on one strand (hemi-methylated as Gm5CNGm5C, complement strand GCNGC), while others (e.g., NhoI and SqiI) cleave the hemi-methylated oligos poorly (Xu et al., 2016). To study BisI activity on modified duplex oligos with one to four m5C and different positions of the modified cytosines in the GCNGC site, we constructed nine sets of modified oligos as shown in the schematic diagram (Figure 8A). There are nine different methylation patterns in this sequence including variants with one, two, three or four m5C (the central nucleotide was always unmodified). Three of the combinations are palindromic where the location of the modified m5C in the upper strand corresponds to the pattern in the bottom strand. Three other variants represent hemimethylated sites and the last three sites have asymmetric methylation patterns with methyl groups distributed between the two strands.

Figures 8B,C show the incubation time dependence in BisI digestion. The experimental data were processed as indicated in Materials and Methods and the percentage of the hydrolyzed product was plotted vs time of the incubation.

The oligo-digestion results indicated that the best substrates are oligos with two internal m5C (1^∗^/1c in Figure 8B), and oligos with three m5C including two central ones (1^∗^/3c and 3^∗^/1c). Oligos with two m5C (one internal and second one external) may be considered intermediate substrates because one strand was cleaved well (1^∗^/2c), but a second strand (2^∗^/1c) was cleaved two times more slowly. The poorest substrates are oligos with two external m5C (2^∗^/2c) and oligos with only one external m5C (2^∗^/4c and 4c^∗^/2). Surprisingly, hydrolysis of the oligonucleotide duplex 3^∗^/3c in which all four cytosines are methylated shows significantly different kinetics from the standard curves of cleavage of other oligo duplexes (Figure 8C). The initial cleavage rate of this substrate is faster than other modified oligos. i.e., approximately half of the fully methylated oligos were cleaved in the early phase and quicky reached a plateau, but the remaining substrate was not further digested after a longer incubation time. We suspect that after the initial fast cleavage of the modified duplex (3^∗^/3c), BisI formed a stable enzyme-product complex by tight binding and would not dissociate from the cleavage products to participate in the second round of hydrolysis. This phenomenon was only observed for the fully methylated duplex with four m5C. Substrates with three m5C (1^∗^/3c and 1c^∗^/3) were cleaved efficiently by BisI endonuclease. As expected, BisI did not cleave N4mC-modified oligo duplex (data not shown).

The ability of BisI to cleave a recognition sequence with only one m5C is a unique property of this enzyme. We were unable to detect cleavage of a recognition site with one m5C during studies of BisI homologs such as BlsI, GlaI and KroI (Chernukhin et al., 2007, 2011; Tarasova et al., 2008) (SKD, unpublished result). Due to this property of the enzyme, BisI may be used to nick at any GCNGC site in a long DNA substrate. For this purpose, an oligo with the internal sequence Gm5CNGC, which is complementary to the DNA sequence of interest, may be used to form a specific complex with a target DNA. With the addition of BisI, this complex can be cleaved with the formation of a nick in the complementary GC↓NGC sequence of the target DNA. The methylated oligonucleotide is specifically designed to ensure the cleavage at the desired site (Supplementary Figure 4A).

In a proof-of-principle experiment, we carried out hydrolysis of plasmid pUC19 at one of the 19 GCNGC sites, located at position 254–258 nt. Modified oligos P1 and P2 (see Materials and Method) were mixed with pUC19 pre-linearized with *Dri*I and the hydrolysis reaction was carried out as indicated in Materials and Method. Supplementary Figure 4B shows the results of BisI cleavage of linearized plasmid pUC19 with two complementary methylated oligos. Hydrolysis of pUC19 was observed only in the presence of both methylated oligonucleotides with formation of two DNA fragments with lengths corresponding to the calculated ones (1,242 bp and 1,444 bp). This experiment demonstrated the ability of BisI to hydrolyze a plasmid DNA at a particular GCNGC site of interest. Presumably the use of a hemi-methylated oligo (Gm5CAGm5C) with two m5C may increase the cleavage efficiency.

## Discussion

There are over 160 BisI homologs listed in the REBASE with significant sequence similarity to BisI. The predicted DNA recognition sequences are GCNGC, RCNRC, or GCWGC with m5C (Roberts et al., 2015). We characterized a number of BisI homologs in this work with unique enzyme properties (e.g., thermostability) or different enzyme specificities.

In addition to McrBC, MspJI, PvuRts1I, and EVE-HNH family REases, two pairs of REases are available to interrogate m5C-modified sites, one cleaving unmodified sites and the other cleaving m5C-modified sites such as *Fnu*4HI (GCNGC) and BisI (GCNGC, two to three m5C); CasI (GCNNGC) and Esp638I/Pam7902I (GCSSGC, two to four m5C) (15). If particular CpG site methylations are important for turning off enhancer function in the regulatory region of oncogenes, the m5C-dependent REases could be useful tools to study epigenetic gene regulation and cancer development. The recognition sequence of Esp638I and Pam7902I potentially overlap with a maximum of four CpG dinucleotides (cGCGCGCg).

A number of BisI homologs (e.g., KpnW2I and VarFI) were found to exhibit partial DNA nicking activity on unmodified DNA. We suspect such relaxed specificity (star activity) might be useful in attenuation of phage infection by frequent nicking of invading DNA or killing self to prevent phage maturation from the infected cells. The double activity on unmodified DNA and m5C-modified DNA blurs the boundary between Type II and IV restriction systems. However, the possible *in vivo* star activity of KpnW2I and VarFI remain to be investigated in the native hosts where star activity is likely to be influenced by ionic strength and availability of divalent cations (Mg^2+^ or Mn^2+^). For the Type II *Kpn*I restriction system it has been shown that the WT enzyme, which has a strong star activity provided better protection (phage attenuation) against phage infection because the enzyme has more target sites to attack and is thus able to reduce the number of phage progeny (Vasu et al., 2012). It was noted before that PvuRts1I family enzymes (glucosylated-hm5C > hm5C > m5C > > C) also displayed strong star activity in digestion with excess enzymes. The dual activity on hm5C and glucosylated-hm5C provides the ability to restrict phage T4gt and T4 (Janosi et al., 1994). The GmrSD REase also has dual activity on both glucosylated-hm5C and hm5C-modified phage DNA, but dependent on ATP or GTP hydrolysis (He et al., 2015).

We found three active BisI homologs from metagenome sequences derived from deep-sea vent and hot spring. Since the genes were from thermophilic sources, we expected to find thermostable BisI homologs. Indeed, one enzyme is active up to 65°C and the other two are active up to 50°C. The thermostable REases may be useful in cleaving modified DNA and in isothermal amplification of certain sequences from mammalian genomes if the somatic mutation eliminated a Gm5CNGC site (i.e., preferential amplification of the mutant DNA while the WT sequence is cleaved before and during the amplification process).

BisI does not cleave the unmethylated DNA sequence GCNGC, nor the symmetrically methylated sequence G4mCNGC (4mC = N4mC) but if at least one cytosine in any position within this sequence is methylated (m5C), the modified DNA can serve as a BisI substrate. The best substrates for BisI include oligos with two internal m5C (Gm5CNGC) and oligos with three m5C including two central ones (Gm5CNGm5C). Oligos with two m5C in complementary GC-dinucleotides (Gm5CNGC) and oligos with only one internal (Gm5CNGC) are substrates for intermediate cleavage efficiency. Oligos with one external m5C are poor substrates for BisI. Oligos containing four m5C shows initial fast cleavage, but did not proceed to completion, suggesting tight binding of enzyme to the cleavage products and unable to turn over. Poor cleavage of heavily modified phage DNA (T4gt and Xp12) by BisI homologs had been reported before (Xu et al., 2016). This observation is reminiscent of certain Type IV REases that do not have apparent cleavage activity *in vitro*, but are able to attenuate phage infection *in vivo* by tight binding to modified invading DNA (Czapinska et al., 2018). In a proof-of-concept experiment, we demonstrated that BisI may be used to partially cleave a designated GCAGC site in a plasmid using methylated oligos specifically designed to target the desired sequence. It is anticipated that hemi-methylated oligos with two m5C (Gm5CAGm5C) may further enhance the cleavage efficiency of such a duplex.

*Dpn*I endonuclease activity on modified DNA: The structure of *Dpn*I endonuclease in complex with N6mA-modified DNA had been solved. *Dpn*I contains the N-terminal catalytic domain with the PD-(D/E)xK motif and the C-terminal winged helix (wH) domain for m6A binding (Mierzejewska et al., 2014). A hydrophobic pocket is formed by residues Leu129, Arg135 and Trp138 in the wH domain, which are all located in a long loop that becomes ordered upon specific DNA binding. Trp138 plays a critical role in the recognition of the modified bases (m6A) in both strands. It simultaneously interacts with both methyl groups: its pyrrole ring is in van der Waals contact with the m6A of the proximal DNA strand and its benzene ring with the m6A of the distal strand (Mierzejewska et al., 2014). Mom modification (methyl-carbamoyladenine modification or ncm^6^A) by phage Mu DNA in Dam^+^ background can still be cleaved by *Dpn*I, suggesting mom modification did not interfere with Dam methylation and *Dpn*I recognition (Hattman, 1979). Other 5hmdU-derived modifications in phage DNA, such as *Salmonella* phage ViI (Vi1) with 5-(2-aminoethoxy)methyldeoxyuridine (5-*N*e*O*mdU) and *Delftia* phage phi W-14 (ΦW-14) with α-putrescinylthymidine (putT) are resistant to *Dpn*I digestion; Overall ViI and phi W-14 are resistant to 70.0% and 68.8% of Type II restriction enzymes *in vitro* restriction digests (Flodman et al., 2019). Recently, a three gene cluster encoded enzymes are shown to be responsible for generating 2-aminoadenine (base Z) modified DNA in cyanophage S-2L and Vibrio phage PhiVC8 which is resistant to large number of Type II restrictions (Sleiman et al., 2021; Zhou et al., 2021). *Dpn*I is unable to cleave PT-modified sites (GpsATC, ps or PT for phosphorothioate DNA backbone modification).

Other MDREs and enzyme engineering: The Gm5C target recognition domain (TRD) or specificity determinants may be utilized in nature to create new specificity by gene duplication or insertion into other DNA binding elements. More recently, the EVE protein domain in the PUA superfamily has been found in fusion with the HNH nuclease domain to provide restriction of hm5C/m5C-modified DNA (Lutz et al., 2019). EVE domain can be fused with PD-D/E xK nuclease and other nuclease domains such as Pin nuclease, PLD nuclease, and GIY-YIG family nucleases (Bell et al., 2020). SauUSI is another example of Type IV REases with PLD nuclease-DNA helicase (ATPase)-Sra domain fusion that restricts hm5C/m5C modified DNA (Xu et al., 2011; Tumuluri et al., 2021). It appeared that in nature, both MDREs with defined cleavage site (e.g., BisI and *Dpn*I) and those with undefined cleavage distance (McrBC, SauUSI, and GmrSD) have evolved in multiple occasions. Studies of both type of MDREs will provide information and strategies to engineer new specificities. Only a few successful specificity engineering efforts have been reported for MDREs: PvuRts1I variants with enhanced activity toward hm5C (Shao et al., 2014) and ScoMcrA variants with relaxed binding activity to PT modified sites (GpsGCC/GpsATC/GpsAAC, ps or PT, phosphorothioate DNA backbone modification) (Yu et al., 2020), and *Bam*HI variant which prefers to cleave GGm6ATCC modified sites (Whitaker et al., 1999). The MspJI family enzymes LpnPI, *Asp*HI, and SgrTI cleave the DNA sites Cm5CDG (D, A/G/T, not C), YSm5CNS (Y, C/T; S, G/C), and Cm5CDS respectively. In the LpnPI crystal structure, Loop-6C and Loop-2B serve as separate DNA binding determinants. A double-swap LpnPI variant ‘LpnPI-27HTG-91RLL’ containing the AspBHI Loop-6C and the SgrTI Loop-2B generated a more relaxed LpnPI variant that cleaves the Sm5CDS site (Gm5CDG and Cm5CDC) (Sasnauskas et al., 2015). However, such specificity module swapping failed to generate active MspJI variants (m5CNNR), suggesting minor differences in enzyme structure can hinder such specificity engineering efforts (Sasnauskas et al., 2015).

## Data Availability Statement

The datasets presented in this study can be found in online repositories. The names of the repository/repositories and accession number(s) can be found in the article/Supplementary material.

## Author Contributions

SX, EZ, DG, ZS, PW, and AF provided experimental data. SX, SD, and RJR designed experiments. SX and SD wrote the manuscript. RJR performed final editing. All authors contributed to the article and approved the submitted version.

## Conflict of Interest

SX, PW, ZS, AF, and RJR are employed by New England Biolabs, Inc. SD, EZ, and DG are employee of SibEnzyme Ltd. (Russia). The authors declare that this study received funding from New England Biolabs, Inc., and SibEnzyme Ltd. The funders were not involved in the study design, collection, analysis, interpretation of data, and the writing of this article. The funders encouraged to submit this work for publication.
