# Supplementary material for: Characterization of BisI Homologs
Source: Front Microbiol. 2021 Jul 1;12:689929. doi: 10.3389/fmicb.2021.689929 (PMC8281217; doi:10.3389/fmicb.2021.689929)
Supplement: Supplementary file 1 [file Data_Sheet_1.docx]

**Supplement Materials: Suppl. Figures S1-4.**

**Characterization of BisI homologs**

Shuang-yong Xu^1^*, Elena V Zemlyanskaya^2^, Danila A Gonchar^2^, Zhiyi Sun^1^, Peter Weigele^1^, Alexey Fomenkov^1^, Sergey Kh Degtyarev^2^*, and Richard J Roberts^1^

1. New England Biolabs, Inc. 240 County Road, Ipswich, MA 101938, USA.
2. SibEnzyme Ltd. 2/12, Ak. Timakova Street, Novosibirsk-117 630117, Russia.

* Corresponding authors:

Dr. Shuang-yong Xu, [xus@neb.com](mailto:xus@neb.com), telephone: 1-978-380-7287

Dr. Sergey Kh Degtyarev, [degt@sibenzyme.com](mailto:degt@sibenzyme.com), telephone +7-9139114907

**Suppl. Fig. S1. Schematic diagram of BisI and BisII restriction systems, and the orphan methylase M.BisIII encoded in the *B. subtilis* T30 genome.** The C5 methylase gene next to BisI (ORF shown in blue) is probably a pseudogene. BisII is a Type I restriction-modification system consisting of *hsdM*, *hsdS*, and *hsdR* genes. M.BisIII is an orphan methylase with Cm5CWGG specificity. The GenBank accession number for BisI genome: CP011051(4,031,727 bp).

**
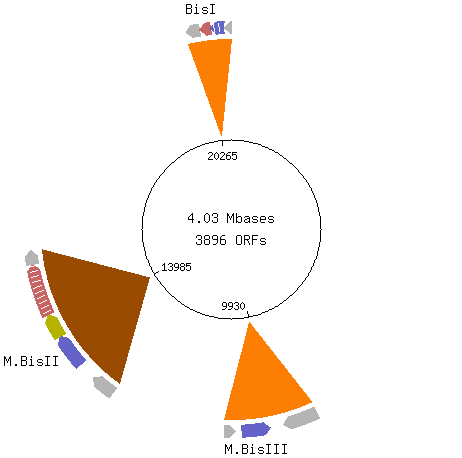
**

**Suppl. Fig. S2. Location of prophage sequences in the *B. subtilis* T30 genome using PHAST (**[**http://phast.wishartlab.com/**](http://phast.wishartlab.com/)**).** Four prophage regions were identified: of which one region is intact (37.4 kb, *Bacillus* phage SPBc2, shown in a red box), three regions are incomplete.

**
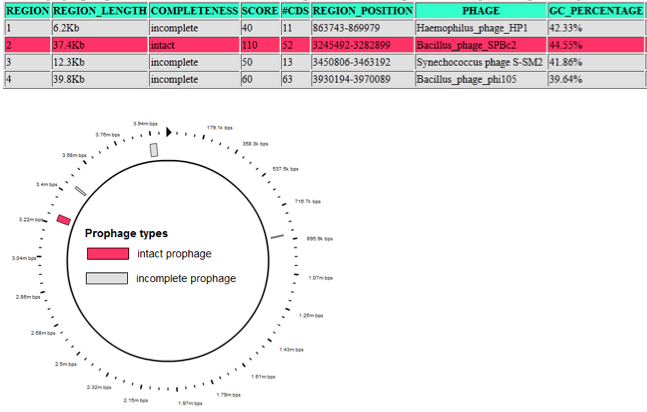
**

**Suppl. Fig. S3.**  **BisI homolog sequences derived from metagenomes of hot spring and deep-sea vent.** BisI homologs #1-#3 are active and thermostable. BisI homolog #4 failed to produce protein for activity assay.

1. **BisI homolog 1** = **EsaHLI** (metagenome source: hot spring)

Synthetic gene sequence

ATGCCCGATCTTGCCCAACTGACAGCCGCTGAGCTTCTGGCGCTTCATGCTCAAATAGGAGAAGAGTTAAGAACGCGCGG

CGTTGTTCGCAGTGCAAATAACCCCACAGGAGATCTGGCAGAACACCTTTTTTGCGCGGCTTTTGGATGGGCTCAAGCTC

CTAACAGCGAACGCGGGTACGATGCAACAGGGCCTGACGGCACTAGATTTCAGATCAAAGGACGTCGGGTACATCGCCGT

AACCCCTCAAGACAGCTTTCTGCGATCAGAGATTTAGCCGGTGGACATTTCGACGTTTTGGCAGGTGTCATCTTCGATGA

CGATTTTTGTGTTGTGAGAGCGGCCCTTATACCTCGGGGAGTGGTCGAGGCTCGTTCTACCTATGTAGCTCATACCAACA

GTCACAAATTTATATTGCGTGAAGACGTCTGGTCCGCGCCTGGGGTACGTGACGTTACCGCCGAAGTCGCAGCTGCGATG

CCT

Amino acid sequence (161 aa residues, 98% sequence identity to SqiI sequence)

MPDLAQLTAAELLALHAQIGEELRTRGVVRSANNPTGDLAEHLFCAAFGWAQAPNSERGYDATGPDGTRFQIKGRRVHRR

NPSRQLSAIRDLAGGHFDVLAGVIFDDDFCVVRAALIPRGVVEARSTYVAHTNSHKFILREDVWSAPGVRDVTAEVAAAM

P

1. **BisI homolog 2** = **EsaTMI** (metagenome source: deep-sea vent, Esa2768829)

Synthetic gene sequence

ATGGATGAGGTGGTCGACTTAGGATCATTGAAAACTAATGAAATCATAAGCCTTTATTCTGACGTGATGGTGGAACTTAA

AAAGCGGGACGTCATCCGCACAAAGAATTTAATAGGGGACCTTGGAGAATATCTTGCTATCAATCATTACAATAACACCA

GCAACCTGCCTAATTTGCGTGAAGCGGCAGTAGGCACGAAAAATATAGATGCCATTTCGCGCGACGGGGACAGATACAGT

ATTAAATCAACCACAGGAAAATTAACTGGAGTATTTTACGGGTTAAACAGTCCAGACTCCGAGGATACAGACAGCCAGAA

ATTCGAGTTCCTGATCATCGTGATTTTTGATGATGAGTTCCGGTTGCACAAAATTTTAGAAGTTTCGTGGGAATTATTCT

TGAAGTACAAGCGGTGGCATACAACAATGAATGCCTGGAATATCTCCATTACGAAAAAACTGACCTCGGAGGCAGTCTGC

ATCTATCAGAGA

Amino acid sequence (164 aa residues)

mdevvdlgslktneiislysdvmvelkkrdvirtknligdlgeylainhynntsnlpnlreaavgtknidaisrdgdrysiksttgkltgvfyglnspdsedtdsqkfefliivifddefrlhkilevswelflkykrwhttmnawnisitkkltseavciyqr

1. **BisI homolog 3** = **EsaGBI** (metagenome source: deep-sea vent, Esa1668319)

Synthetic gene sequence

ATGGACTTGAAAGACTTGGATGATGAGGAGTTAGTCAACCTTTACCCTGAGTTACTTAAAGAATTGAAGAACCGGGATAT

AATTAGAACGAACAACCTTGTAGGAGAACTTGGTGAGTATCATGCGCACCGGATTTACAAAAAAGACACGAGCCTGCCCC

AATTACAACTGAATTTAAAAAGCACAAAAAACATTGACGCAACGAGCGTCAGAGGCGAACGCTATGCCATTAAAAGTACC

AGCGGAAATGGTACGGGAGTCTTCGCGTCGCTGCCTAAGGTAGATGACGGTGTAGTCCATTTTGAGTACCTGATTATAGT

AATATTCAACAAGGATTACACCGTTAAAGGGATCTTTGAGTTATCTTGGAAACAGTTTCTTGAGTTCCGCAAAATGAAGC

AGCCAGAGAACAAATGGAATGTACCCATAACGTCATCGTTGAAGAAGTCGGCTAAGATAATACTT

Amino acid sequence (155 aa residues)

MDLKDLDDEELVNLYPELLKELKNRDIIRTNNLVGELGEYHAHRIYKKDTSLPQLQLNLKSTKNIDATSVRGERYAIKST

SGNGTGVFASLPKVDDGVVHFEYLIIVIFNKDYTVKGIFELSWKQFLEFRKMKQPENKWNVPITSSLKKSAKIIL

1. **BisI homolog 4** (Esa1772744) (failed to produce sufficient amount of protein)

Synthetic gene sequence

ATGGAGATATTCGACAAATTTAAATCCTTAACTGTACAAGAACTTGCCGAACAGATATCGCCATCGTCTAGTGCGTTCAT

CGAATTAAAAAAGCGCGGAATTTTAAGAACGAAAAATGTAGTTGGGGAGCTGGGAGAATACTTCGCAGTCCAATACTACA

ACGATAATTCAAAACTGCCGAACCTGAGCCTTGCCCCGCCTGGAGTAAAGAATATAGATGCACTTTCCAGAGATGGGGAG

ATATACAGCATAAAGACAGTCTCGTCCCGTTCAGGCACCACCGGTAGCTTCTGGGACCCAGAGTCTATACAGAATAACAT

CAAGAAGTTCGATTATTTAGTGATCGTCATATTAAATAACTCTTATTCCGTAGACGTAGTCTTGCAAATGACGTGGAATG

ATTTTCTTGAGAACAAACGGTTCAATAGCCGTATGAATAATTACAACATCTCTGTGACAAAGAAACTGATCACCGAGTTT

CATATC

Amino acid sequence (162 aa residues)

MEIFDKFKSLTVQELAEQISPSSSAFIELKKRGILRTKNVVGELGEYFAVQYYNDNSKLPNLSLAPPGVKNIDALSRDGE

IYSIKTVSSRSGTTGSFWDPESIQNNIKKFDYLVIVILNNSYSVDVVLQMTWNDFLENKRFNSRMNNYNISVTKKLITEF

HI

**Suppl. Fig. S4.** **Targeted BisI cleavage of pUC19 (pre-linearized with DriI).**

**A.** Schematic diagram of m5C-modified oligos annealing to a DNA target followed by BisI digestion. **B.** BisI digestion of linearized plasmid with annealed modified oligos (Gm5CAGC and Gm5CTGC). Lanes 1 and 6, 1 kb SE DNA Ladder; lane 2, pUC19 DNA (DriI cut); 3. pUC19 (DriI) + BisI; 4. pUC19 (DriI) annealed to methylated oligos; 5, pUC19 (DriI) annealed with methylated oligos + BisI. Two partially digested products were detected.
